# Superior hypogastric plexus block – Transdiscal approach

**DOI:** 10.1016/j.inpm.2026.100802

**Published:** 2026-07-01

**Authors:** Kelly Li, Michael Erdek, Eileen T. Jin, David Hao

**Affiliations:** a Harvard Medical School, Boston, MA, USA; b Northwell Health Cancer Institute, New York, NY, USA; c Department of Anesthesiology, Mass General Brigham, Harvard Medical School, Boston, MA, USA

This video reviews the fluoroscopy-guided superior hypogastric plexus block using a trans-discal approach. The superior hypogastric plexus block is an established intervention for visceral pelvic pain, most commonly in patients with gynecologic, colorectal, or genitourinary malignancies, as well as in select refractory nonmalignant pelvic pain syndromes. In the trans-discal technique, the needle is advanced through the L5–S1 intervertebral disc to access the retroperitoneal space containing the superior hypogastric plexus.

The superior hypogastric plexus is a bilateral retroperitoneal structure located at the level of the aortic bifurcation, typically at the L5 to S1 level. It consists predominantly of sympathetic fibers arising from T12 to L2 via the aortic plexus and lumbar ganglia, with minor parasympathetic contributions. Inferiorly, the plexus divides into the right and left hypogastric nerves, which descend into the pelvis and contribute to the inferior hypogastric plexus.

This video reviews indications, key anatomic landmarks, and the step-by-step fluoroscopy-guided trans-discal technique.

The primary indication for a superior hypogastric plexus block is intractable visceral pelvic pain. This is most commonly related to malignancy, including gynecologic, colorectal, and genitourinary cancers. The procedure may also be considered in chronic pelvic pain conditions such as endometriosis, pelvic inflammatory disease, or postsurgical adhesions when pain is visceral in origin and refractory to pharmacologic therapy.

The procedure begins with an anteroposterior fluoroscopic image obtained at the L5–S1 level, aligning the superior endplate of S1. The C-arm is then adjusted using a combination of ipsilateral obliquity and cephalocaudal angulation to visualize a working corridor into the pelvis. This alignment creates a window bounded by the superior articular process, the iliac crest, and the superior endplate.

Through this window, a short introducer needle or 18-gauge spinal needle may be used to traverse the dermis. A long 22-gauge spinal needle is then advanced through the disc and beyond the anterior annulus fibrosus into the retroperitoneal space. A curved needle may be used to facilitate guidance and potentially reduce disc trauma by enabling completion in a single pass.

Nonionic contrast, such as iohexol, is injected to confirm appropriate retroperitoneal distribution, ideally demonstrating patchy spread, and to exclude intravascular, sub–anterior longitudinal ligament, or, less commonly, intrathecal placement. Both anteroposterior and lateral fluoroscopic views should be used to confirm accurate spread.

After negative aspiration, a diagnostic block may be performed using a local anesthetic, commonly 1% lidocaine or 0.25% bupivacaine. If neurolysis is indicated, dehydrated alcohol (typically 98%) or phenol (commonly 6%) may be injected slowly, generally in a total volume of 10–15 mL. Local anesthetic in an equal volume should be administered before injection of the neurolytic agent, particularly when using alcohol. Continuous patient monitoring is essential during this phase.

Potential adverse effects include hypotension related to sympathetic blockade, discitis, nerve root injury, transient back or abdominal pain, transient bowel or bladder dysfunction, and local infection or hematoma. Meticulous technique, contrast confirmation, and post-procedural monitoring are critical to risk mitigation.

## Declaration of interest

We have no conflicts of interest to report.

